# Clinical doses of radiation reduce collagen matrix stiffness

**DOI:** 10.1063/1.5018327

**Published:** 2018-04-03

**Authors:** Joseph P. Miller, Brandon H. Borde, Francois Bordeleau, Matthew R. Zanotelli, Danielle J. LaValley, Dylan J. Parker, Lawrence J. Bonassar, Susan C. Pannullo, Cynthia A. Reinhart-King

**Affiliations:** 1 Meinig School of Biomedical Engineering, Cornell University, Ithaca, New York 14853, USA; 2 Department of Biomedical Engineering, Vanderbilt University, Nashville, Tennessee 37235, USA; 3 Sibley School of Mechanical and Aerospace Engineering, Cornell University, Ithaca, New York 14853, USA; 4 Department of Neurological Surgery, New York Presbyterian Hospital/Weill Cornell Medical Center, New York, New York 10032, USA

## Abstract

Cells receive mechanical cues from their extracellular matrix (ECM), which direct migration, differentiation, apoptosis, and in some cases, the transition to a cancerous phenotype. As a result, there has been significant research to develop methods to tune the mechanical properties of the ECM and understand cell-ECM dynamics more deeply. Here, we show that ionizing radiation can reduce the stiffness of an *ex vivo* tumor and an *in vitro* collagen matrix. When non-irradiated cancer cells were seeded in the irradiated matrix, adhesion, spreading, and migration were reduced. These data have ramifications for both *in vitro* and *in vivo* systems. *In vitro*, these data suggest that irradiation may be a method that could be used to create matrices with tailored mechanical properties. *In vivo*, these suggest that therapeutic doses of radiation may alter tissue mechanics directly.

## INTRODUCTION

Radiation therapy is used on almost half of cancer patients as a method to cause cell death by damaging DNA.^1^ The effects of radiation on cell death are well established, but less is known about the effects of radiation on the extracellular matrix (ECM) residing within and surrounding tumors.

Several studies have been performed to investigate the effects of ionizing radiation (IR) on the cellular microenvironment; however, much of this research uses models where the radiation is applied to a cellularized environment.^2–5^ In these cases, the effects of the radiation on the ECM and the effects of the radiation on the cells (which then alter the ECM) become difficult to isolate.

The matrix within solid tumors is markedly different from the matrix within healthy tissue. During tumor progression, increased extracellular matrix (ECM) deposition and cross-linking result in increased tumor stiffness.^6–9^ Changes in the stiffness of the tumor microenvironment have been shown to be a contributing factor in cancer malignancy and metastasis.^10–12^ In light of these findings and others, there is significant interest in understanding how matrix stiffness changes during tumor progression, the effects of tissue stiffening on cells, and the development of therapeutics to inhibit or reverse stiffening.^13^

Here, we investigated the effects of ionizing radiation (IR) on collagen. Our data indicate that IR reduces the stiffness of both non-cellularized collagen scaffolds and *ex vivo* mammary tumors from MMTV-PyMT transgenic mice. Infrared spectroscopy and confocal imaging were employed to analyze the chemical and architectural changes, respectively, and notably, we found that while stiffness is decreased, collagen fiber architecture was not significantly altered. Cell adhesion, cell spreading, and cell migration all decreased in irradiated matrices, in line with what is expected from cells interacting with a less stiff matrix. These data indicate that radiation reduces matrix stiffness, suggesting that radiation may be a novel method to manipulate matrix mechanics *in vitro* and *in vivo*. This work helps to expand our understanding of radiation biology and may have a significant downstream impact on patient treatment strategies.

## RESULTS

### Mechanical testing of irradiated mammary tumors

**FIG. 1.**
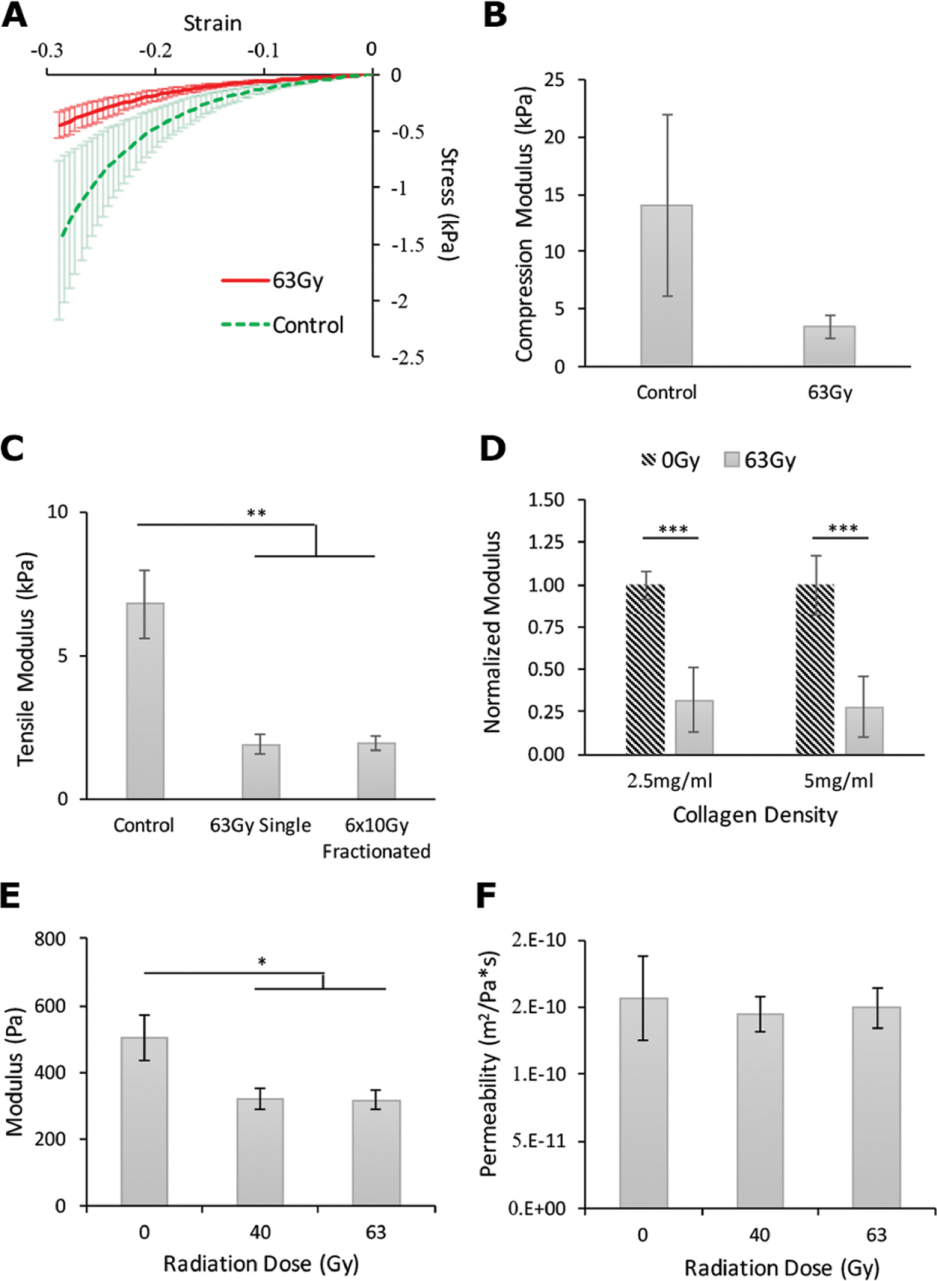
Effects of IR on matrix mechanical properties. (a) Stress/strain compression curves indicating a difference in the slope (modulus) between tumors treated with 63 Gy (Red, n = 6) and untreated (Green, n = 4) across a range of strains (Wilcoxon signed-rank test with continuity correction: ^***^p < 0.001). (b) Compression testing of mammary tumors between untreated (14 080 ± 7917 Pa) and their pairs irradiated at 63 Gy (3452 ± 972 Pa). (c) Tensile testing of *in vitro* collagen scaffolds revealed no difference between single fraction (1880.90 ± 820.74 Pa) and multi-fraction doses (1938.50 ± 528.07 Pa) (n = 10, ^**^p < 0.01) but significantly lower modulus compared to control (6784.85 ± 3746.99 Pa). (d) Normalized tensile modulus showing the effect of radiation to be significant in both 2.5 mg/ml (n = 10, 0.32 ± 0.19) and 5 mg/ml (n = 10, 0.28 ± 0.18) collagen gels (p < 0.001). (e) Compressive testing (n = 10, ^*^p < 0.05) showing a reduced modulus in the treatment groups (40 Gy: 322.75 ± 99.12 Pa; 60 Gy: 317.51 ± 87.62 Pa) compared to control (504.85 ± 217.25 Pa). (f) Permeability (n = 10) of the collagen scaffold following treatment showed no significant change.

Increased stiffness of the tumor ECM is due in large part to additional collagen deposition and increased crosslinking by the cellular population during disease progression.^9,10^ To test whether irradiation alters tumor stiffness, tumors were extracted from mice, immediately irradiated, and tested via mechanical compression. The results revealed that stiffness is significantly reduced for irradiated tumors compared to untreated tumors and increasingly so with increased strain rates [Fig. 1(a)]. This result corresponds to the previously published literature of Mohamed *et al.*^2^

### Mechanical testing of irradiated 3D collagen scaffolds

Motivated by the results of the animal tissue experiments, but recognizing the convolution of the complex tissue with the effects of radiation, *in vitro* 3D collagen matrices were used to investigate the impact of the radiation on the mechanical and structural properties of the primary ECM protein, collagen. To determine if the effects exhibited a dose-rate dependency, we tested both single-dose and fractionated dose treatments. Fractionated radiotherapy delivers a large total dose of radiation through consecutive, smaller doses separated by time. It is currently the standard of care for many cancer treatment plans.^23^ Thus, the tensile modulus of 5 mg/ml collagen irradiated in a single fraction of 63 Gy was compared with 6 doses of 10 Gy separated by 24 h and untreated gels (control). In the case of single-dose treatments, the modulus decreased in the collagen gels. Similarly, in the case of the fractionated-dose treatments, the modulus decreased to an approximately equivalent level [Fig. 1(c)]. It is important to note that the radiation source produces only monoenergetic photons such that the total energy imparted to the collagen gels is identical in both treatment cases. Together, these results suggest that the stiffness-reducing effect of radiation is dose-dependent, not rate-dependent, and therefore a function of only total exposure time to the photon source.

We next investigated whether the modulus-reducing effect was dependent on collagen density. Both 2.5 mg/ml and 5 mg/ml collagen gels were irradiated and underwent tensile testing as these densities are regarded as the most significant for breast cancer research.^7^ Data from each treatment group were normalized against its respective control group in an effort to better display the relative reductions in moduli due to the radiation treatment. The results indicate that the modulus of both densities of collagen was significantly decreased compared to their non-irradiated controls [Fig. 1(d)] by approximately the same percentage. Thus, the stiffness-reducing effect of IR on a collagen matrix is independent of the matrix density.

Collagen is a viscoelastic material which exhibits mechanical behaviors not detectable using tensile testing alone. Thus, confined compression testing on 5 mg/ml collagen gels irradiated at 40 Gy and 63 Gy was also performed. Again, the treatment groups showed a reduction in matrix stiffness compared to controls [Fig. 1(e)]. The difference between the treatment groups was not significant and less than the changes in tensile modulus relative to their control. This weaker response can be explained by noting the geometry of any given fiber in the matrix. With the small aspect ratio (diameter/length) of a collagen fiber, the stretching (tensile) mode will be significantly stiffer compared to compression, where the dominant fiber behavior will be bending.^24^

### Ionizing radiations effect on collagen microarchitecture

To investigate whether radiation affects the porosity and microstructure of the collagen matrix, we measured the permeability of the irradiated gels based on the diffusion of liquid from the gel under step-wise compression. Importantly, no significant changes in the rate of this diffusion were detected [Fig. 1(f)]. Since the measure is dominated by the porosity of the gel under compression, the lack of difference between irradiated gels and controls implies an insignificant change in matrix architecture and that the softening of the collagen matrix is not simply due to the destruction of the matrix.

**FIG. 2.**
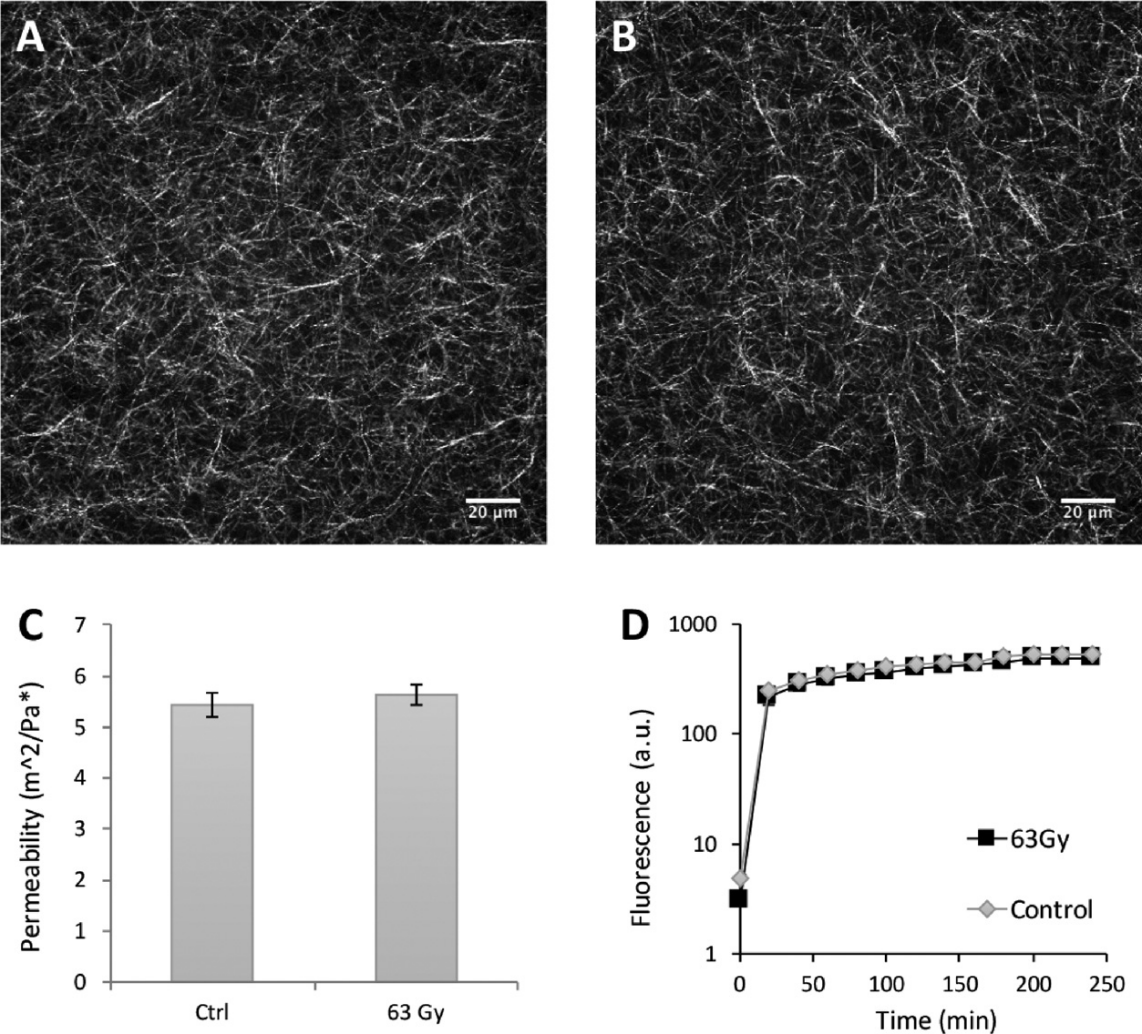
Quantitative analysis of matrix architecture. Confocal reflectance images (a) before and (b) after irradiation. Scale bars are 20 *μ*m. (c) Characteristic pore size determined by autocorrelation of control gels (5.43 ± 1.26 *μ*m) and irradiated gels (5.63 ± 1.13 *μ*m) (n = 9). (d) Permeability (Log-scale) of the FITC assay showing no significant change in the rate of diffusion between treatment and control gels (n = 3).

Maintaining the density of the collagen matrix constant, confocal reflectance microscopy was used to visualize collagen fiber architecture following radiation treatment. There were no significant architectural changes in gels before [Fig. 2(a)] or after 63 Gy of radiation [Fig. 2(b)]. To quantify the porosity of the matrix, autocorrelation studies on “before” and “after” confocal reflectance images were performed. The results indicate that radiation had no significant impact on the pore size of matrices [Fig. 2(c)]. This result supports the data indicating that there were no significant differences in hydraulic permeability [Fig. 1(f)]; since the pores are of the same size, the outflow remains unchanged. To determine whether any media or chemical diffusion differences were present in the treatment group, we performed a permeability assay using a Fluorescein isothiocyanate (FITC) dye. The resulting diffusion curves show no significant differences in diffusion rates [Fig. 2(d)]. All together, these results indicate that radiation decreases matrix stiffness without significantly altering matrix architecture.

### Infrared spectroscopy of irradiated collagen scaffolds

After determining that radiation decreases the modulus of collagen without significantly altering its architecture, we investigated the mechanism by which irradiation alters stiffness. Fourier Transform Infrared (FT-IR) spectroscopy was utilized to investigate the chemical landscape of the irradiated collagen.

**FIG. 3.**
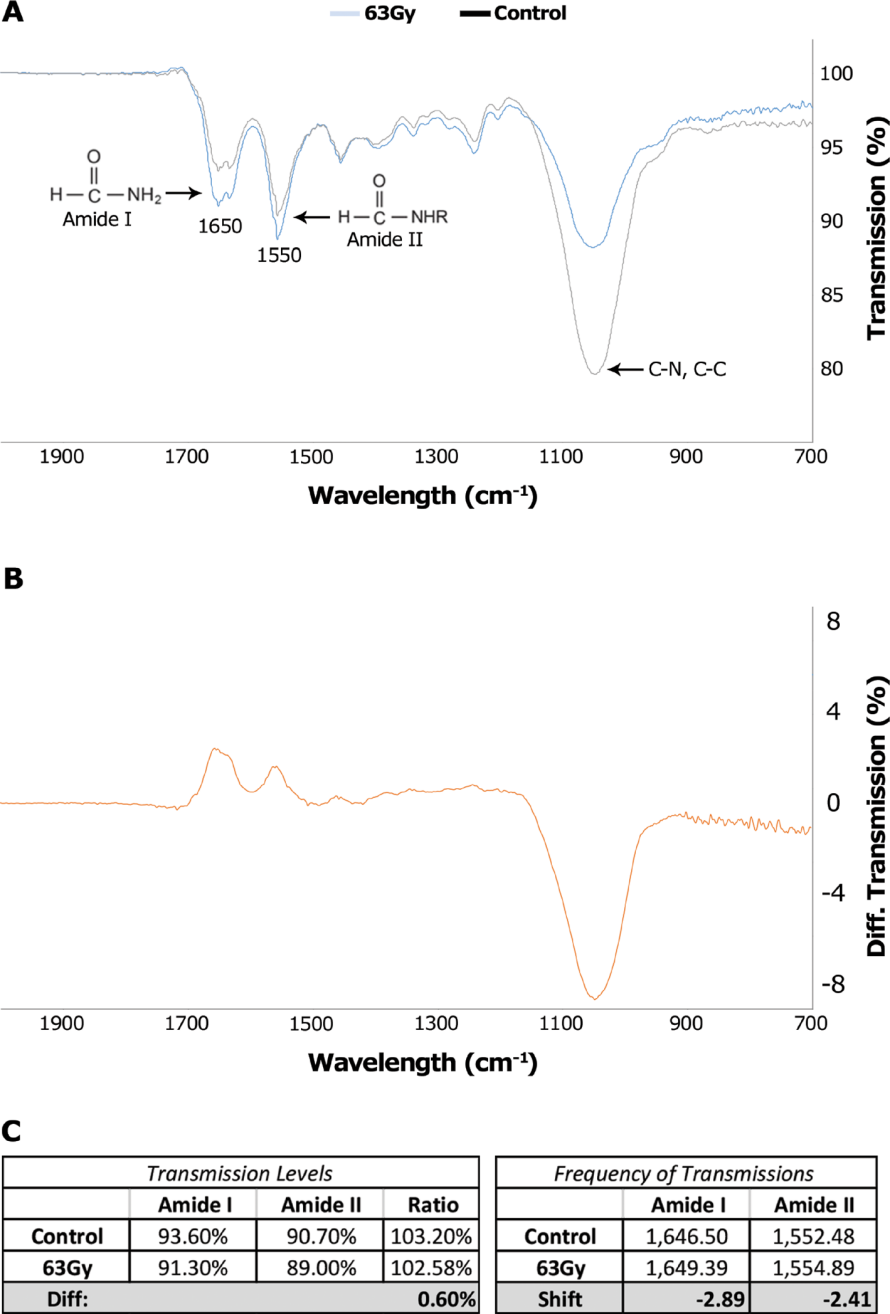
IR spectra of irradiated and control collagen. Irradiated collagen matrices show a reduction in the 900–1200 cm^−1^ region, indicating a reduced number of C-C and C-N bonds. The presence of consistent Amide group I and II ratios and peak locations between control and treatment groups indicate that the collagen helix has not been denatured.

FT-IR spectra of irradiated versus control collagen gels revealed a dramatic reduction in the 900–1200 cm^−1^ fingerprint region, indicating a lower presence of C-C, C-O, and C-N bonds (Fig. 3). The C-N and C-C bonds form the backbone of the collagen protein. The presence of both the 1640 cm^−1^ Amide I and 1550 cm^−1^ Amide II regions, both of which are characteristic of the collagen spectra,^25,26^ indicates that the collagen has not been denatured by the radiation. It is important to note that the ratio of these peaks is not significantly changed by the treatment (0.6% difference). In addition, there is a small shift in both peaks; however, this shift is within the device's level of precision. It is believed that the helical folding of collagen is stabilized by hydrogen bonds between the NH residues from peptide bonds of one chain and the carbonyl groups of an adjacent chain, and this formation is enabled by the fixed angle of the C-N peptidyl-proline.^27^ Thus, it is possible that radiation-induced cleavage of these peptide bonds would destabilize the collagen triple helix by disrupting the helical primary structure.

### Cell adhesion and spreading

Changes in matrix stiffness are expected to alter cell behavior.^28,29^ To investigate the effects of irradiation-induced softening, highly metastatic, non-irradiated MDA-MB-231 cancer cells were seeded on irradiated collagen gels, and the cells were monitored for adhesion, spreading, and invasion.

**FIG. 4.**
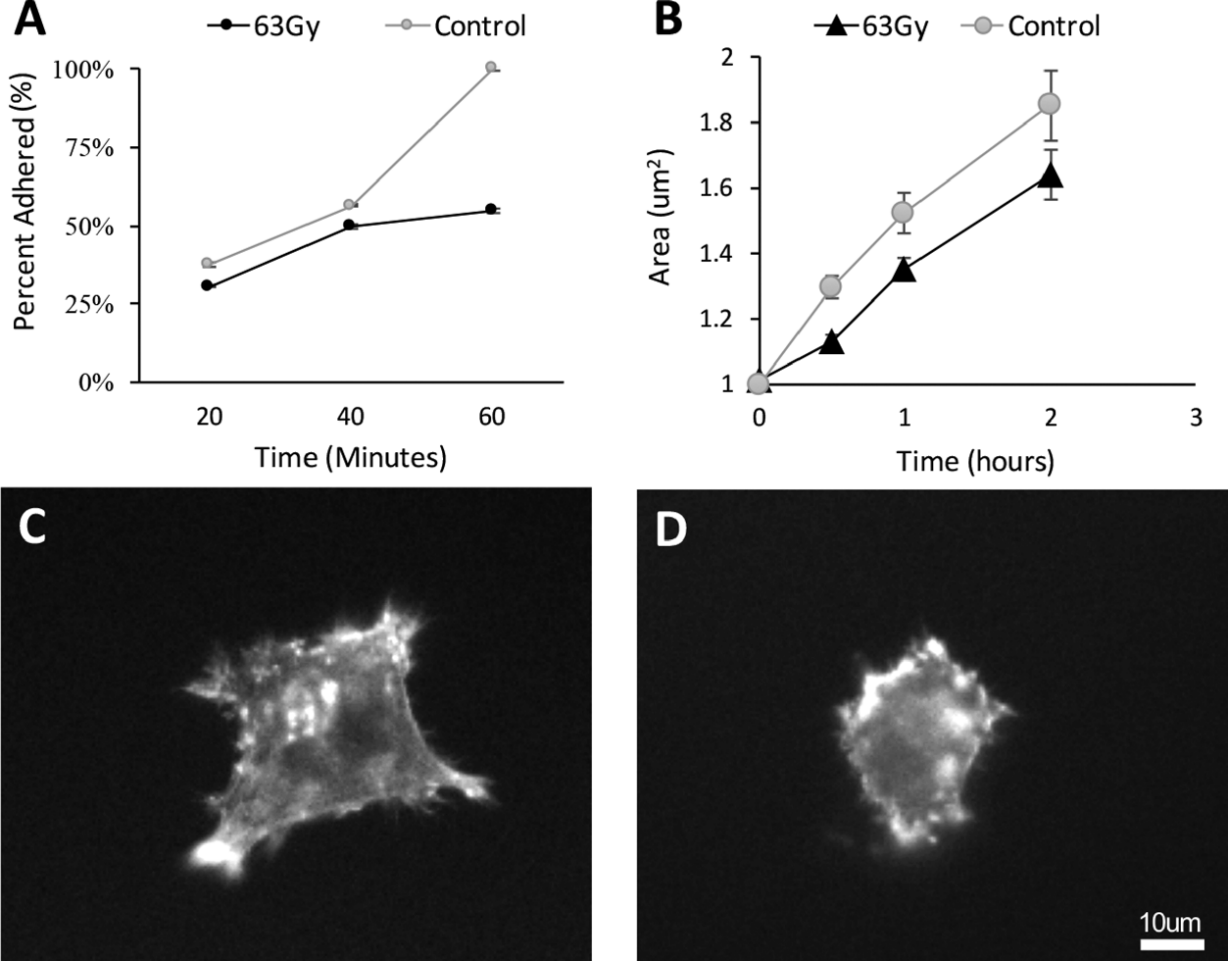
Cell adhesion over time. (a) MDA-MB-231 cells exhibited a reduced capacity to adhere to irradiated collagen gels (n = 354) compared to controls at 60 min (n = 508). (b) The cell rate of spreading is also reduced on the softer irradiated collagen gels (n = 586) compared to controls (n = 451) (Control Cell Area = 75.20t, R^2^ = 0.98; Irradiated Cell Area = 58.44t, R^2^ = 0.99) (n = 6). Fluorescence image of the representative cell on (c) control gels compared to (d) cells on irradiated gels at 2 h post-plating.

Over a 60 min period, cells exhibited a reduced capacity for adhesion to irradiated collagen compared to controls [Figs. 4(a)–4(c)]. Similarly, cell spreading over time revealed the same trend. Cells that were seeded on the less stiff, irradiated matrices exhibited difficulty in initial spreading and exhibited consistently smaller areas throughout the 2 h window within which they were observed [Fig. 4(d)].

### Cell invasion

**FIG. 5.**
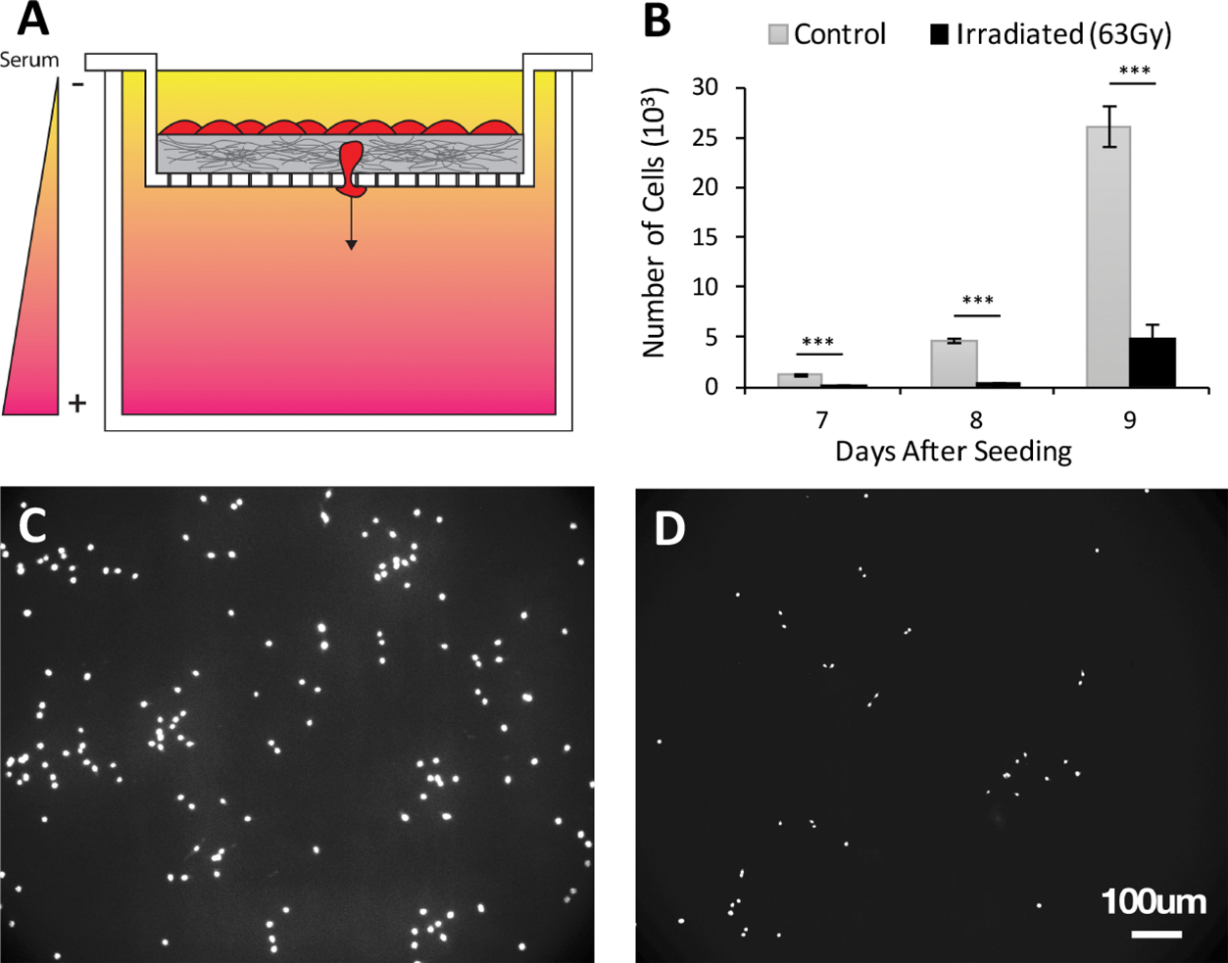
Cell invasion over time. (a) Transwell invasion schematic with serum gradient conditions. Cells are seeded on 500 *μ*m of 2.5 mg/ml collagen and allowed to invade through the matrix and pass through 8 *μ*m pores. (b) Invasion results indicating that cells invade through irradiated collagen at a persistently lower rate compared to controls. (n = 6). Representative fluorescence images (DAPI) of invaded cells through (c) control gels and (d) irradiated gels.

To determine the effects of ECM-targeted radiation on cell invasion, a transwell invasion assay was performed [Fig. 5(a)]. After 7 days, fewer cells invaded through the irradiated collagen gels compared to untreated gels, consistent with the behavior of cells in less stiff collagen.^9,30,31^ Further, after 2 days of additional invasion, consistently fewer cells were capable of traversing the matrix as revealed by fluorescence images of 4′,6-Diamidino-2-Phenylindole (DAPI) stained cells [Figs. 5(b)–5(d)].

## DISCUSSION

The work described here indicates that ionizing radiation can reduce the stiffness of a 3D collagen matrix and cells respond to the more compliant matrix with decreased adhesion, spreading, and migration. FT-IR spectroscopy confirmed that radiation does not denature the collagen and revealed that the reduction in stiffness occurs, at least in part, due to the cleavage of C-C and C-N peptide bonds in the backbone of the collagen protein. This was a key finding supporting the hypothesis that radiation adds a unique capability to the suite of methods aimed at controlling the ECM mechanical properties. Overall, these results demonstrate that IR can effectively lower a 3D collagen matrix stiffness without significantly altering the architecture, thus allowing for a more controlled study of cell-ECM dynamics.

While our data indicate that IR reduces the stiffness of collagen, we did not investigate the compound effects of irradiating both the matrix and the cells within the matrix. Understanding how radiation might impact epithelial cells, fibroblasts, immune cells, as well as other cell populations in the cancer environment is critical in determining whether the mechanical effects reported here have potential for clinical impact. It is well known that even single doses on the order of 1 Gy have the potential to significantly alter cell behavior.^32^ As a result, future work must be done to understand which micro-dosing schedule, if any, allows for the mechanical reduction to occur over a time period that is sufficiently long to allow cells to recover from any deleterious effects of the micro-dose while still affecting the ECM mechanical properties. Certainly, if such a dosing schedule does exist, the results presented here suggest the potential to move radiation from its exclusive use of targeted cell death into an entirely new regime of manipulating the mechanical properties of the ECM for the purposes of cancer treatment and prevention.

We utilized confocal reflectance imaging to confirm the architecture of the collagen before and after irradiation; however, the technique is not without limitations.^33^ While our data indicate that there are no structural changes in collagen that are visible at the resolution of optical imaging, it is possible that smaller scale changes exist. Additional investigation using Scanning Electron Microscopy (SEM) can be used to further explore the mechanisms of the radiation/collagen interactions.

Finally, our results raise an interesting possibility with respect to two current questions in radiobiology: namely, why do some cell lines appear more radio-resistant than others, and why do certain radiation-based treatment plans have more or less favorable clinical outcomes.^34–39^ The results presented here raise the possibility that the effects of IR on the mechanical properties of the microenvironment may have a significant impact on the net outcome of radiation treatment plans via changes to cell-ECM interactions. Interestingly, it has been shown that cancer cells can be sensitized to various drugs by altering the stiffness of the ECM.^36,37^ Together, these data suggest that there may be a IR-ECM-drug efficacy space that has the potential for clinical impact. At a minimum, in addition to understanding how various cell types respond to radiation directly, future research must also investigate how cells respond to the mechanical changes within their environment induced by irradiation of the ECM. The latter effect very well may prove to be a principle component in determining downstream effects on patient outcome.

## METHODS

### Collagen preparation

Collagen was harvested from Sprague-Dawley rat tail tendons as previously described.^14,15^ Briefly, tendon bundles were removed and suspended in a 0.1% acetic acid solution at 150 ml/g tendon. The mixture was allowed to sit at 4 °C for at least 48 h and then centrifuged at 9000 RPM for 90 min. The supernatant was collected, frozen, and lyophilized for 48 h or until all water was removed. Stock solutions containing the lyophilized collagen reconstituted in acetic acid were prepared at 6 mg/ml and stored at 4 °C until use. Working solutions containing 10× Dulbecco's Phosphate Buffered Saline (DPBS), 1 N NaOH, and 1× Dulbecco's Modified Eagle's Medium (DMEM; Life Technologies, Grand Island, NY) were prepared and mixed with stock solutions, resulting in gels at 2.5 and 5 mg/ml collagen density. Each gel was then allowed to polymerize at 37 °C for 30 min.

### Mechanical testing

Collagen gels at a thickness of 2 mm were prepared and irradiated at the doses described (clinically relevant doses of 10 Gy to 63 Gy) using a Mark I Model 68 Cesium Gamma Irradiator. Tensile dog bone punches were taken from each sample with a gauge length of 5 mm. Each tensile sample was tested to failure at a strain rate of 3%/s. The resulting load data were analyzed for the tensile modulus by least squares regression over the elastic region of the curve. Compressive samples were taken from irradiated collagen gels using a 6 mm circular biopsy punch. Each sample was tested under confined stress relaxation compression to 30% strain in increments of 5%. Using a custom MATLAB program (Mathworks, Natick, MA), a poroviscoelastic model was fit to the data and analyzed for gel equilibrium and instantaneous stiffness, as well as hydraulic permeability.^16^

Harvested mammary tumors were tested in compression on a uniaxial Enduratec ELF3200 load frame (Bose Electroforce, Eden Prairie, MN) to 30% and 50% strain at a rate of 2%/s. The resulting stress and strain were calculated from the resulting load-displacement data in the same fashion as the collagen gels.

### Confocal reflectance and fluorescence microscopy

Confocal fluorescence and reflectance images were acquired as described previously^17^ using a Zeiss LSM700 confocal microscope on a Zeiss Axio Observer Z1 inverted stand equipped with a long-working-distance water-immersion 40×/1.1 numerical aperture Zeiss objective. Fluorescence labeling and imaging of actin and nucleus (DAPI) were performed, and ImageJ (version 1.49b, National Institutes of Health, Bethesda, MD) was used to quantify the actin area and cell count via DAPI-stained nuclei from confocal fluorescence images as previously described.^18^ For quantitative characterization of the collagen scaffold architecture, we measured the scaffold pore size using autocorrelation as described before.^15^ Briefly, the autocorrelation function was fitted with a Gaussian curve, and 1/e^2^ was used to estimate the pore size diameter.

### Permeability assay

Collagen gels were transferred into ThinCert™ polyethylene terephthalate (PET) membrane 6-well cell culture inserts with a pore size of 0.8 *μ*m (Greiner Bio-One) and incubated for 45 min at 37 °C for polymerization. Treatment gels were then given a dose of 63 Gy. For the assay, 1 ml of 10 *μ*M 40 kDa FITC-dextran (Sigma, St. Louis, MO, USA) in complete media was added to the upper chamber, while 2 ml of complete media was added to the lower chamber. 100 *μ*l samples were collected from the lower compartment, placed into a glass bottom 96 well-plate, and then replaced with an equal volume of complete media every 20 min for 4 h. A 200 *μ*m z-slice of each sample was captured using a Zeiss LSM700 confocal microscope on a Zeiss Axio Observer Z1 inverted stand equipped with a long-working-distance water-immersion 40×/1.1 numerical aperture Zeiss objective at 488 nm. Fluorescence intensity was then measured using ImageJ from a 150 × 75 *μ*m “Region of Interest” (ROI) approximately 10 *μ*m from the bottom of each well.

### FT-IR spectroscopy of irradiated collagen scaffolds

1 mg/ml collagen samples were analyzed using Fourier transform infrared (FT-IR) spectra obtained from a Thermo Scientific Nicolet iS10.^19–21^ Backgrounds were acquired from the DMEM culture media used to make the collagen as described earlier. Scans were obtained at a resolution of 4 cm^−1^ between 700 and 2000 cm^−1^ and reported as the average of three scans for each condition.

### Cell culture

Highly metastatic MDA-MB-231 breast adenocarcinoma cells, validated through short tandem repeats (STR) (Catalog No. HTB-26, American Type Culture Collection, Manassas, VA), were maintained in Dulbecco's Modified Eagle Medium (DMEM; Life Technologies, Grand Island, NY) supplemented with 10% (vol/vol) fetal bovine serum (FBS; Atlanta Biologicals, Flowery Branch, GA), 100 U/ml penicillin (Pen Strep), and 100 g/ml streptomycin (Life Technologies). All cell culture was maintained at 37 °C and 5% CO_2_. Cells were trypsinized and passaged with 0.25% trypsin-EDTA (Mediatech) 1:4 every 2 days or until growth to 80% confluence. All cell studies were performed between passages 10 and 15.

### Cell adhesion and spreading

Cell adhesion and spreading studies were performed on 2.5 mg/ml collagen. 100 cells/cm^2^ were seeded on top of untreated or irradiated collagen gels. After being allowed to attach and spread for the indicated time, cells were washed 3 times with 1× phosphate buffered saline (PBS) and fixed with 3.7% formaldehyde in PBS for 15 min. Cells were stained for DAPI or phalloidin-Alexa Fluor^®^ 564 and imaged through 35 mm plastic petri dishes. The acquired images were analyzed using ImageJ.

### Cell invasion

Cell invasion studies were performed in a 6-well plate with a transwell insert containing 8 *μ*m pores. A 500 *μ*m layer of collagen was polymerized on top of the transwell membrane. 5000 cells/cm^2^ were seeded on top of the collagen. 1 ml of serum-poor media (DMEM with 5% FBS and 1% Pen Strep) was placed in the transwell. Outside of the transwell, contained by the well plate, 2 ml of serum-rich media (DMEM with 10% FBS 1% Pen Strep) was placed. The serum gradient was replaced every 48 h for 7 days. After 7 days, the bottom of the transwell was treated with 0.25% Trypsin and rinsed twice with 1× PBS to remove adherent cells. The well plate was then fixed with 3.7% formaldehyde PBS for 15 min and stained with DAPI. Fluorescence images were taken and quantified using an ImageJ particle analyzer. The transwell was then placed in a new 6-well plate, and the serum gradient is reestablished. This process was repeated every 24 h for two additional days.

### Mice

All mice were maintained following a protocol approved by the Cornell University Institutional Animal Care and Use Committee (Assurance #A3347–01). MMTV-PyMT transgenic mice from the FVB strain background were obtained from the Jackson Laboratory. No mice were excluded from the study. Pairs of tumors were extracted and irradiated as control/treatment pairs. No blinding was performed. Mammary tumors were excised from 10 to 12 week old female MMTV-PyMT transgenic mice. The freshly isolated mammary tumors were immediately irradiated, then flash frozen in liquid nitrogen before mechanical testing, and later thawed as described previously.^22^

### Code availability

All algorithmic analysis was performed using the default standards with FIJI (ImageJ) and can be acquired from open-source http://imagej.net/Fiji/Downloads.

### Statistical methods

All significance calculations were performed from student t-tests unless otherwise noted. Tests for normality were applied to validate the application of the t-test. In addition, all data are reported as mean ± standard errors. All *in vitro* experiments were performed in independent triplicates (r = 3) unless stated otherwise.
